# Formation of Nanocompounds of TiO_2_ Using PVA-HAp Nanofibers by Sol-Gel Technique

**DOI:** 10.3390/polym17202796

**Published:** 2025-10-19

**Authors:** Marvin Elco Estrada Macias, Humberto Alejandro Monreal Romero, Guillermo Martínez Mata, Rosaura Pacheco Santiesteban, Claudia López Meléndez, Héctor Alfredo López Aguilar, Oscar Chávez Acosta, Carlos A. Martínez-Pérez, Caleb Carreño-Gallardo, José Guadalupe Chacón-Nava

**Affiliations:** 1 Department of Biomaterials Science and Nanotechnology, University of Chihuahua (UACH), Avenue University, Chihuahua 31000, CHIH, Mexico; meestrada@uach.mx (M.E.E.M.); gmata@uach.mx (G.M.M.); rpacheco@uach.mx (R.P.S.); 2 Department of Engineering and Materials, La Salle University, Avenue Lómas de Majalca 1120, Chihuahua 31625, CHIH, Mexico; clopez@ulsachihuahua.edu.mx (C.L.M.); hlopez@ulsachihuahua.edu.mx (H.A.L.A.); 3 Postgraduate Coordination, Instituto Tecnológico de Chihuahua II, Chihuahua 31130, CHIH, Mexico; oscar.ca@chihuahua2.tecnm.mx; 4 Institute of Engineering and Technology, Autonomous University of the City of Juarez, Ciudad Juárez 32310, CHIH, Mexico; camartin@uacj.mx; 5 Advanced Materials Research Center, S.C. (CIMAV) and National Nanotechnology Laboratory, Avenue M. Cervantes 120, Industrial Complex Chihuahua, Chihuahua 31136, CHIH, Mexico; caleb.carreno@cimav.edu.mx (C.C.-G.); jose.chacon@cimav.edu.mx (J.G.C.-N.)

**Keywords:** nanocompounds, polyvinyl alcohol (PVA), nanofibers, sol-gel technique, titanium dioxide, hydroxyapatite

## Abstract

The use of hydroxyapatite (HAp) nanofibers in combination with titanium dioxide (TiO_2_) emerges as a method for the design and improvement of materials at the biomedical, architectonic, and industrial levels. In this research, TiO_2_ nanocomposites were developed using HAp nanofibers through the sol-gel technique. The molecular assembly strategy reveals the formation of nanocomposites with sizes of 100–500 nm at 700 °C. EDS analysis shows the presence of Ca and P, indicating that HAp nanofibers have been integrated into the nanocomposites. The crystalline phases corresponding to rutile and anatase were detected by X-ray spectroscopy analysis. The morphology of the composites was analyzed by surface segmentation analysis, scanning electron microscope, and scanning tunneling microscope.

## 1. Introduction

The use of nanocomposites using various biocompatible materials has gained great relevance in recent years because they exhibit exceptional functional properties. The need to improve physicochemical and mechanical properties, as well as aspects related to corrosion, has led to the rapid emergence of various methods for the synthesis of composites with a wide variety of organic and inorganic molecules. These include titanium, polyvinyl alcohol (PVA), hydroxyapatite, zinc oxide, silicon, tungsten, natural or synthetic polymers, zirconia, concrete, carbon nanotubes, and polymethylmethacrylate [1,2,3,4,5,6]. In this sense, various researchers have studied the photoluminescent characteristics of titanium, such as the case where the photocatalytic degradation of thymol blue and rose bengal was studied using TiO_2_/PAni/GO nanocomposites [7]. The synthesis of TiO_2_ nanopowders and nanostructures has also been explored by various methods, such as sol-gel or hydrothermal processes, to obtain TiO_2_ transducers, photosynthetic sensors for renewable energy, and electrodes to measure electrochemical behavior [8,9,10]. TiO_2_/SiO_2_ composites and TiO_2_-ZnO have been synthesized to degrade organic compounds such as quinolone and as alternatives for antibacterial, antifungal, or anticancer therapies [11,12]. On the other hand, the manufacture of nanoparticles and nanofibers based on materials such as tungsten, modified concrete, and titanium has many applications in various fields of science, such as biomedicine, sustainable architecture, electronics, optics, electromagnetics, and others. Their advantages are related not only to size but also to their morphology and interaction with other materials [13,14]. Other relevant applications of the use of nanofibers are in areas of energy generation and in the solution of various environmental problems. The use of titanium oxide compounds with nanofibers, in the presence of PVA, has had a relevant application for photocatalytic degradation in wastewater contaminated with various pesticides. One of the important aspects of PVA/TiO_2_ nanocomposites is their use in solar cells as anti-reflective coatings, increasing the absorbance intensity and improving thermal stability [15,16]. Likewise, nanocomposites have been developed using hydroxyapatite in the presence of TiO_2_ to evaluate the mechanical properties, compressive strength, and flexibility [17]. Other studies related to the use of hydroxyapatite and TiO_2_ have attracted attention within tissue engineering, in which nanocomposites have been synthesized for bone regeneration through the supercritical CO_2_ method [18]. In this way, hydroxyapatite is a very versatile material in many areas; such is the case in which ion-conducting nanocomposite membranes have been developed using polyvinyl alcohol for applications in fuel cells [19]. PVA-HAp fibers have also been used to evaluate the elongation resistance of bone tissue and for biomimetic studies in hard tissues using electrospinning techniques, improving their thermal properties, as well as surface morphology, pore size, and biodegradation [20,21,22]. There are also several applications of using PVA-HAp nanofibers for vacuum plasma treatments during oxygen delivery in the area of medicine and tissue engineering [4,23,24,25]. With respect to tissue engineering, PVA-HAp composites have been studied in simulated body serum by evaluating their bioactivity and biological crystallization [26]. Other studies show the UV absorption capacity of PVA-HAp nanofibers that have been synthesized in the presence of graphene for the development of air filters for toxic gases and in the generation of composites in the presence of TiO_2_ [27,28]. Furthermore, PVA-HAp-TiO_2_ fiber scaffolds have been proposed as drug delivery pathways in bone tissue regeneration with good osteoconductive properties [29].

The exploration of new techniques for its elaboration has been facilitated by the formation of compounds of TiO_2_ using PVA-HAp, which display unique properties such as low toxicity, mechanical stability, and thermal and chemical stability. Furthermore, a range of molecules and materials, such as cells, fibronectin, alginate, stainless steel, titanium, chromium, and chitosan, has been utilized for this purpose.

The use of metallic materials such as titanium represents an excellent alternative for activities such as medical implants due to their thermal, chemical, and mechanical stability within organic systems. It has been used in various areas, such as immunogenetics, catalysis, polymer science, orthopedic transplantations, etc. Hydroxyapatite, polyvinyl alcohol, and titanium have proven to be very efficient within these areas, since their thermodynamic, electrostatic, and optical properties can increase the molecular recognition of electrostatic interactions with high selectivity in solution. This enhances the stimulus responsiveness of the ionic interaction in supramolecular chemistry processes, with energy dissipation establishing a molecular self-assembly mechanism on the surface of the elements that form part of the system. Additionally, there are different methods in the preparation of nanofibers, such as chemical vapor deposition, electrochemical anodization, electrospinning, hydrothermal/solvothermal method, bottom-up and top-down, template synthesis, sol-gel method, self-assembly method, etc. [30,31,32,33,34]. The synthesis of Ti alloys in the presence of hydroxyapatite and cerium has been reported in numerous cases as biomaterials with a significant ability to inhibit the pathogenic activity of microorganisms and corrosion processes. This accelerates the osseointegration of cerium and HAp during the coating formation process [35]. It is important to highlight the use of TiO_2_, HAp, and PVA in the synthesis of nanofibers due to the several benefits of biocompatibility, innocuousness, and hydrophilic properties, in addition to having various characteristics such as chemical resistance and electrical and mechanical properties, which allow them to be used to generate different supramolecular structures at the nanometer scale. Most of these processes are carried out by molecular recognition, using organic molecules and materials. The resulting molecular structures, in several cases, are assembled to form molecular aggregates and devices with special properties. In this manner, the forces involved are dipole–dipole interactions, solvophobic effects, electrostatic interactions, and hydrogen bonds, among others. In this work, we have synthesized TiO_2_-PVA-HAp nanofiber composites by the sol–gel method. The purpose was to determine the functionality of nanofibers in molecular self-assembly processes in the presence of TiO_2_ molecules.

## 2. Materials and Methods

The research design for the synthesis of TiO_2_-PVA-HAp nanofibers was experimental, using PVA-HAp nanofibers as the independent variable and TiO_2_ as the dependent variable using the sol-gel method as described below.

The synthesis of the TiO_2_-PVA-HAp nanofibers was carried out using the following procedure: 1 g of sodium phosphate was weighed in 100 mL of bidistilled water; then 20 mL of 1 M ammonium hydroxide solution was added. 5 mL of 1 M Ti isopropoxide Ti [OCH (CH3)2]4 −97% (Sigma-Aldrich, St. Louis, MO, USA) were mixed in 11 mL of 99.8% ethanol (Sigma-Aldrich, St. Louis, MO, USA) at room temperature in a flask for the formation of the alkoxide solution. Subsequently, 7 mL of bidistilled water was mixed with 11 mL of ethanol in another flask, and 10 drops of an ammonium hydroxide/sodium phosphate solution at a pH of 7.0 were added with a pipette to form the catalysis solution. The catalysis solution was placed into the alkoxide solution and stirred in a magnetic stirrer for 5 min at a temperature of 37 °C to form the “sol” solution. Then, to form the gel, the sol was placed inside a mold, and the PVA-HAp nanofibers were added. Once the gel had formed, it was placed under ethanol and allowed to age for 24 h at room temperature until the ethanol evaporated. After this time, it was washed 3 times with bidistilled water to eliminate ethanol residues and calcined at 700 °C in a laboratory electrical kiln for 1 h.

### 2.1. Characterization of Nanocompounds by STM

A scanning tunneling microscope Nanosurf Easyscan 2 instrument (Nanosurf Liestal, Switzerland) equipped with Pt/Ir tips (BT00400) at a resonance frequency of 204–497 kHz and a constant force of 10–130 N/m was used to characterize the morphology of the compounds.

### 2.2. Characterization of Nanocompounds by X-Ray Diffraction Pattern

The characterization of the compounds was carried out by means of X-ray diffraction to identify the crystalline phases in a diffractometer Phillips X’PERT X-Ray using a CuK (α) source at 0.1542 nm.

### 2.3. Power Spectral Density Analysis

The analysis of power spectral density (PSD) and Fast Fourier Transform (FFT) was executed utilizing the Mountains Lab 9.0 image processing software using the following parameters: structural analysis, texture direction, control chart, superficial segmentation, and critical dimension analysis.

### 2.4. TGA Analysis

For the characterization of the nanocompounds, a thermogravimetric analysis was carried out using a DTA-TGA TA instrument (New Castle, DE, USA) using a heating rate of 10 °C/min in air.

### 2.5. Coefficient of Determination R2 and Fractal Dimension Analysis

The R2 correlation studies and the fractal dimension were processed using USA version 9 software (Digital Surf, Besançon, France) and Easyscan 2 nanosurf version 1-6-0-0 imaging software.

## 3. Results and Discussion

### 3.1. Characterization of TiO_2_-PVA-HAp Nanocompounds by STM

**Figure 1:**
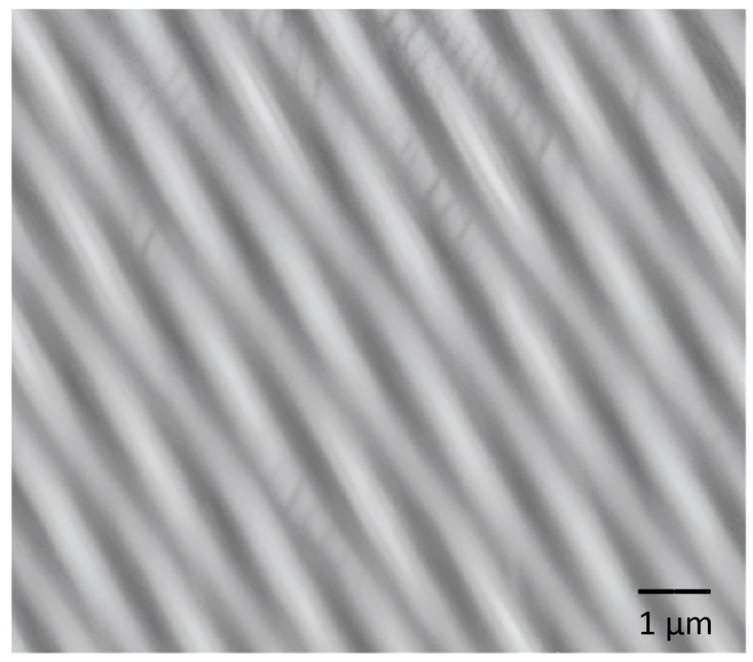
STM of TiO_2_-PVA-HAp nanofibers.

Figure 1 shows the 2D image of the nanocomposites using scanning tunneling microscopy (STM). Clearly defined fibers with diameters of approximately 200–300 nm are observed on the surface. It can be observed that in some parts of the fibers, it presents a depletion in their length. This may be due to the HAp particles playing an important role in preventing the continuous interaction of TiO_2_ during the synthesis due to their agglomeration. The morphology of the fibers has the characteristic of coiling on itself in some parts, leaving a helical shape. This type of morphology is crucial for tissue engineering applications, as it enables the anchoring of various molecules, including proteins, cells, minerals, and other organelles, thereby creating a suitable environment for molecular self-assembly. In other reports, fibers with similar morphology have been reported [36,37].

### 3.2. Thermo-Gravimetric Analysis

**Figure 2:**
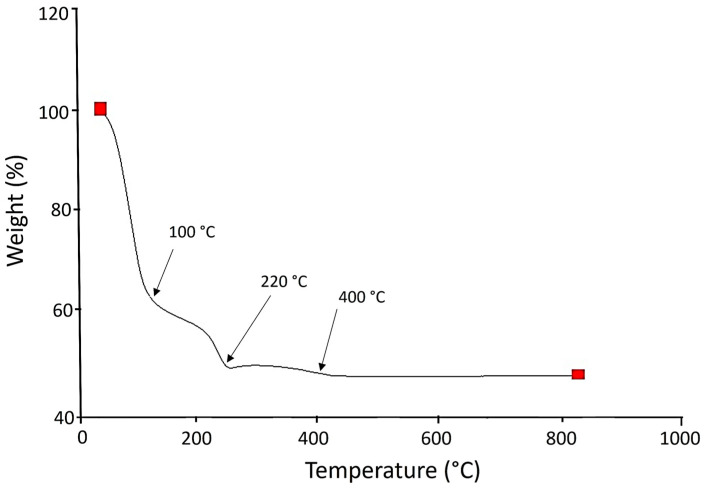
TGA analysis of TiO_2_-PVA-HAp nanocompounds.

Figure 2 is representative of the TGA analysis of the TiO_2_-PVA-HAp composite; a weight loss can be observed at approximately 100 °C and 220 °C, followed by a stabilization of the curve at 400 °C. This result is characteristic of weight loss due to the drying process.

### 3.3. X-Ray Diffraction Pattern

**Figure 3:**
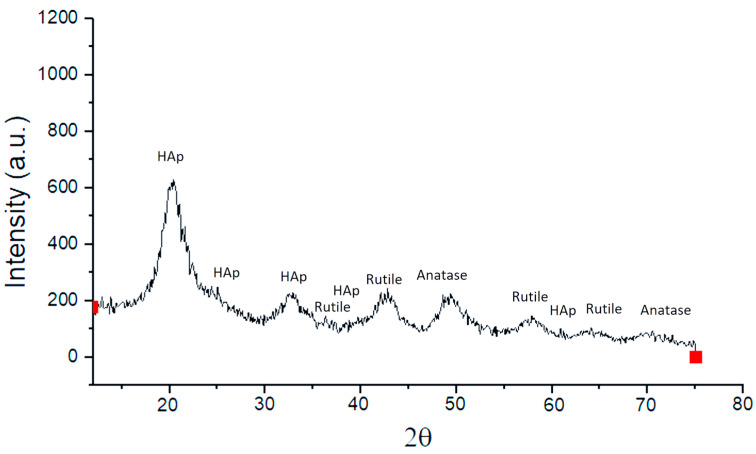
X-ray diffraction pattern obtained from the TiO_2_-PVA-HAp nanocompounds.

Figure 3 shows the X-ray diffraction pattern of TiO_2_-PVA-HAp nanocompounds, showing peaks corresponding to the rutile phase at 36.0°, 44°, 56.7°, and 65° 2θ and peaks corresponding to the anatase phase at 48° and 70° 2θ. The degree of crystallinity was 95% for the rutile phase and 40% for the anatase phase, respectively, and was measured by the intensity of the peaks in the diffractogram. Additionally, a lower intensity can be observed in the peaks at 25.4°, 32°, 38°, and 62° 2θ that correspond to HAp with a degree of crystallinity of approximately 52%. These results are similar to those reported in other works using PVA-HAp nanofiber composite [20]. In addition, there is a high-intensity peak corresponding to HAp at 20°. The compounds obtained have crystalline characteristics and thermal stability of the rutile phase due to the low energy of this phase. On the other hand, the presence of nanofibers in the presence of HAp could participate in the desorption of hydrogen bonds, inhibiting the formation of a large amount of anatase crystalline phase. This avoids destabilization of the electronic configuration of the TiO_2_ and HAp molecules. This is so as not to trigger chain reactions with the consequent formation of the superoxide anion (O_2_) and OH radicals in solution. Additionally, with respect to the short diffraction peaks corresponding to hydroxyapatite, they may be due to an increase or decrease in the red parameters of the unit cell that provide energy to the system. This causes a depletion in the peaks due to the increase in temperature, synthesis method, texture, phase mixture, and size of ions present in the hydroxyapatite. This creates substitutions in the concentrations of calcium and phosphate in the aqueous solution and displacements in the diffraction pattern because the crystals of the sample are oriented in all directions and randomly. In this manner, only some crystals can be oriented correctly to comply with Bragg’s law, corresponding to a set of planes with a certain angle theta. The JCPDS card number is 21-1276.

### 3.4. Average Diameter Analysis

**Figure 4:**
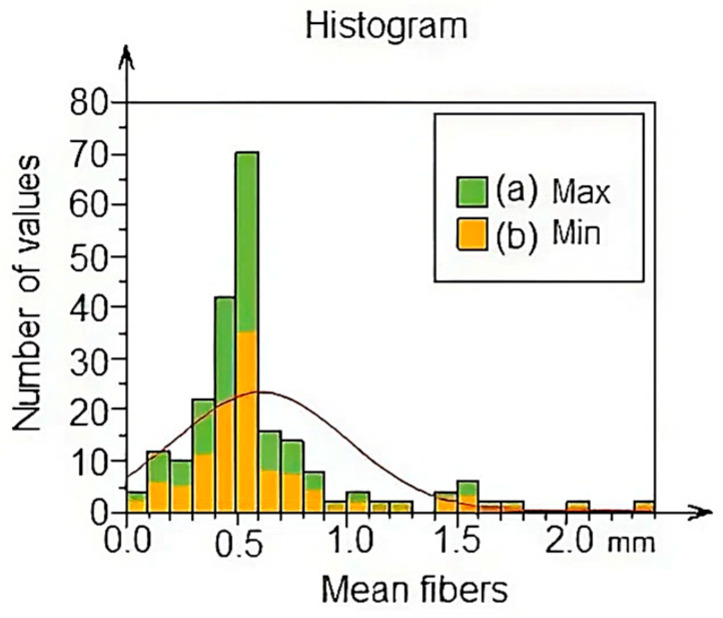
Percentage of the diameter of the fibers TiO_2_-PVA-HAp.

Figure 4 shows a histogram of the percentage of the average diameter of the fibers, showing the maximum values (green) and the minimum values (yellow). It can be seen that the average ranges of the fibers begin at 1 mm and correspond to between 0.2 and 0.49% as minimum and maximum values. It should be noted that the outstanding percentage has a value of 0.6 mm with a range between 0–35% at least and 70% maximum. Also, the lowest percentage is represented by fibers with diameters of 0.0003 mm and a percentage between 1 and 1.5 approximately.

**Figure 5:**
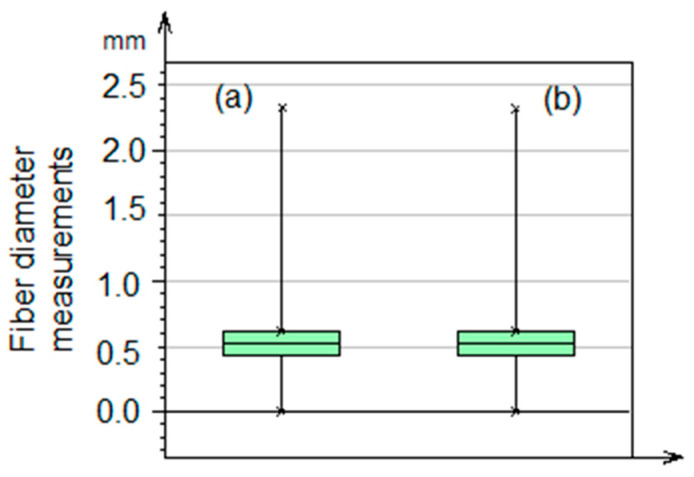
Box diagram: (**a**) mean and median of fibers with maximum value, (**b**) mean and median of fibers with minimum value.

Figure 5 shows the mean and median values of the average fiber diameter using the box diagram. Two vertical lines are similar in the percentage values of the analysis of the maximum and minimum diameter of the fibers corresponding to the median, with a value of 1.25 mm. There is also a cross corresponding to the value of the mean with 0.6 mm. These results are related to the data found in the histogram in Figure 4. Additionally, the standard deviation value was 0.3874 mm.

### 3.5. Characterization of TiO_2_-PVA-HAp Nanocompounds by FFT

**Figure 6:**
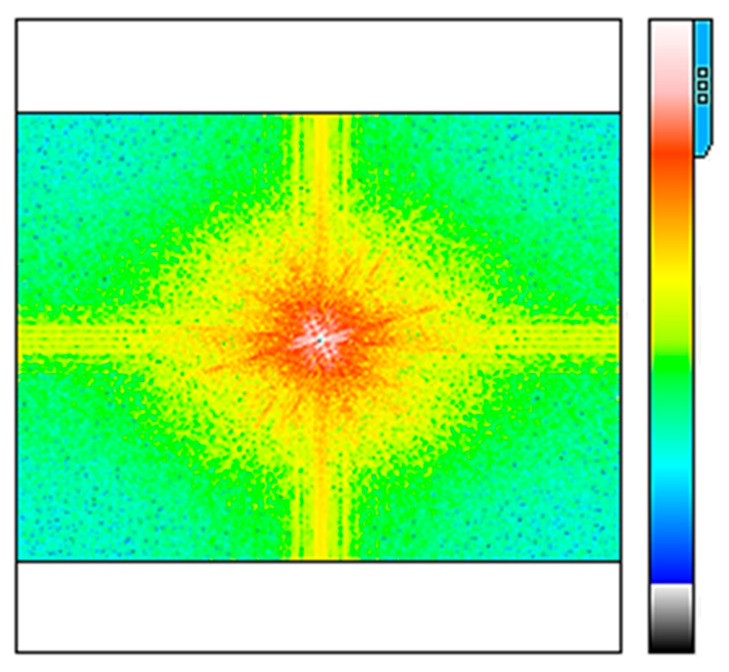
The frequency spectrum of TiO_2_-PVA-HAp.

Figure 6 shows the analysis of the Fast Fourier Transform of the fibers. In this image, it can be seen that there are low-frequency levels located at the center and high-frequency levels as scattered points. This behavior is probably due to electrostatic interactions between titanium molecules and PVA-HAp fibers that can generate variations in wavelength amplitude and frequency across all fibers, which means the existence of great system functionality.

### 3.6. Characterization by Power Spectral Density (PSD) Analysis of TiO_2_-PVA-HAp Nanocompounds

**Figure 7:**
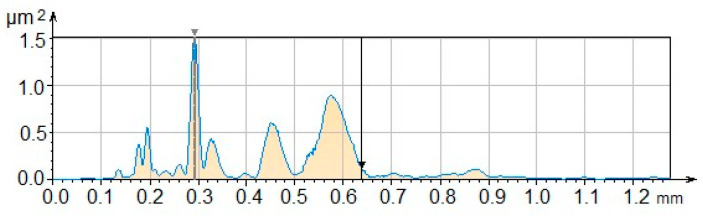
Power spectral density analysis.

The power spectral density analyzes the distribution of the power signal of the fiber frequencies; this analysis is obtained from the results of the Fourier transform, as shown in Figure 6. These results show different levels of periodic signals characteristic of the functionality of the fibers in aqueous medium and how light is dispersed in this environment. Wavelength values are at 0.6388 with an amplitude of 0.3299 µm and a dominant wavelength of 0.2927 mm; see Figure 7.

### 3.7. Coefficient of Determination R2

**Figure 8:**
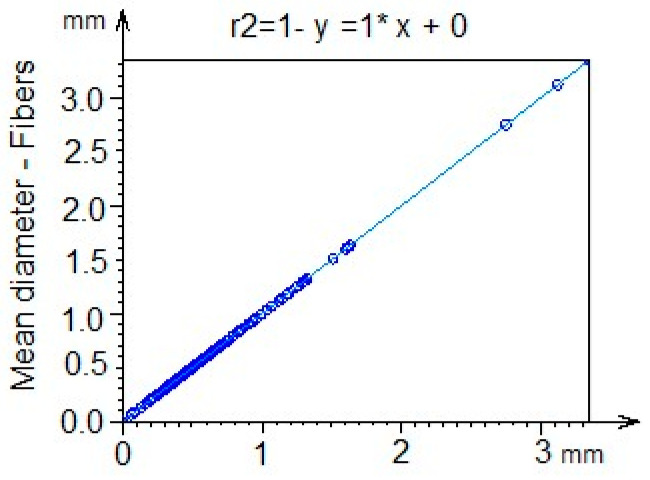
Coefficient of determination (R2) analysis.

To evaluate the replication of the proposed system, an analysis of the coefficient of determination R2 was carried out, which has a value of 1, as shown in Figure 8. These results allow for a prediction of the impact of geometric patterns not defined by the described model through variables such as temperature, pH, and molecular interaction processes, thus ensuring that the method can be reproduced.

### 3.8. Fractal Dimension Analysis

**Figure 9:**
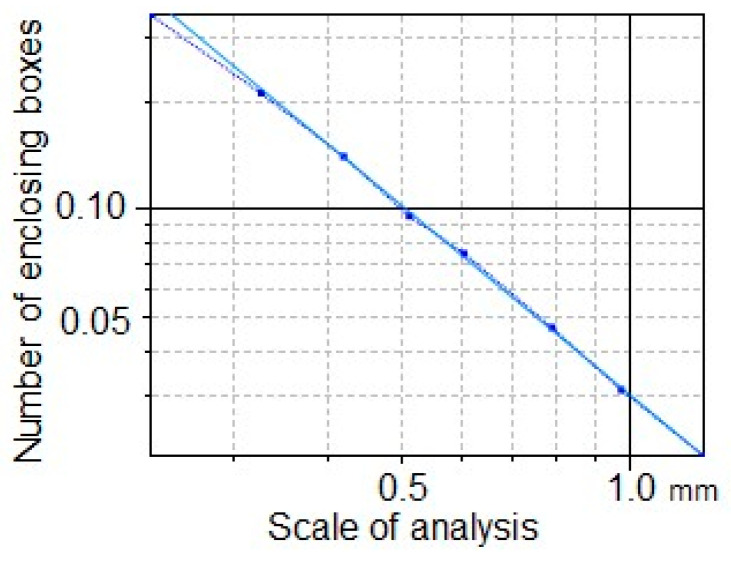
Fractal dimension of TiO_2_-PVA-HAp.

Figure 9 shows the analysis of the fractal dimension with a value of 1.752. The geometric shape of the compounds allows us to establish that it is a dynamic system due to the molecular self-assembly process between the TiO_2_-PVA-HAp nanofibers, allowing for a spatial distribution of morphologically self-similar substructures. The value of the fractal dimension was made using the following equation: log S = D log L → D = log S/log L, where S (8) is the size of the fractal, L is the measurement scale (1.385), and D is the unknown fractal dimension.

### 3.9. Texture Isotropy Analysis

**Figure 10:**
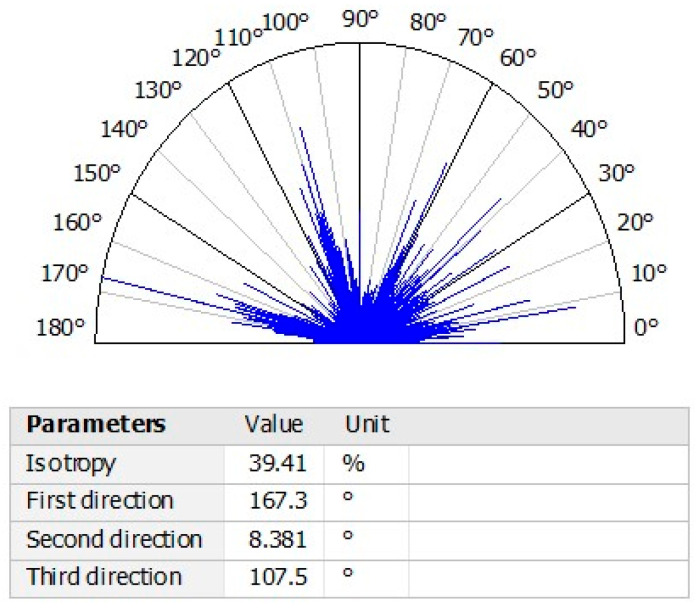
Characterization of nanofibers by surface geometry analysis.

A textural isotropy analysis was conducted to ascertain the directional uniformity parameters of the surface planes of PVA-HAp nanofibers in the presence of TiO_2_. Figure 10 depicts the characteristics of the direction of the surface texture direction, as depicted by the direction of the surface texture direction. The measured values indicated an isotropy of 39.41%, a first direction of 167.3°, a second direction of 8.381°, and a third direction of 107.5°, respectively. These findings demonstrate that the system is isotropic, preserving uniform surface characteristics in all directions. Numerous nanostructured materials, including nanofibers, nanoparticles, nanorods, and nanotubes, have been examined. However, systems such as the one proposed in this work have not been thoroughly examined [38,39]. The angle degrees that correspond to the number of fibers in the sample reveal the position of each point on the surface of the nanofibers. The isotropic texture of the complex matches that of the isotropic texture, since the diverse aggregates that contain the TiO_2_-PVA-HAp are equally distributed.

### 3.10. Characterization of Nanocompounds by Surface Geometry Analysis

**Figure 11:**
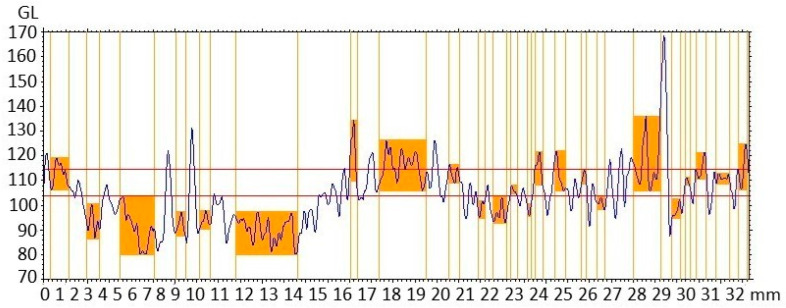
Step height analysis of nanofibers.

Figure 11 depicts an analysis of the surface geometry of the TiO_2_-PVA-HAp nanofibers. It illustrates the profile of the step height of nanofibers with curve-shaped peaks. The values of the step height were between 0.2 and 2.8 mm, with a maximum depth of 16.05 and 34.30 and a medium depth of 13.32 and 24.63 mm, respectively. The geometry of nanofibers has been analyzed using step height in other studies, but not when titanium is present [40,41,42].

### 3.11. Characterization of Nanocompounds by Surface Segmentation Analysis

**Figure 12:**
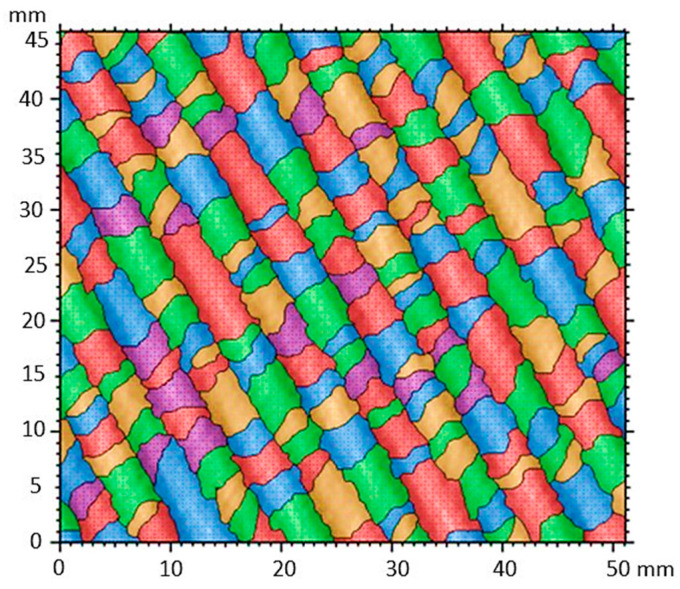
Texture segmentation of nanofibers.

Figure 12 shows the surface segmentation of the texture mapping that accounts for the growth, division, and fusion of regions as patterns of structure recognition. It can be observed that the objects are outlined individually within the image. This segmentation map creates different levels of color, which represent different groups of texture components based on various geometric shapes that make up the nanofibers, such as helices of fibers with small sizes, which surround larger fibers. One can also see how the edges of the fibers are defined along them. The segmentation values relative to the convex area ranged from 0.83 to 1.0 mm^2^, indicating a more prominent fiber center. Furthermore, the line delimiting the fiber contour had a perimeter of 0.2 to 7.8 mm^2^, respectively. These segmentation results are consistent with the texture isotropy analysis shown in Figure 10.

### 3.12. Characterization of TiO_2_-PVA-HAp Nanocompounds by SEM

**Figure 13:**
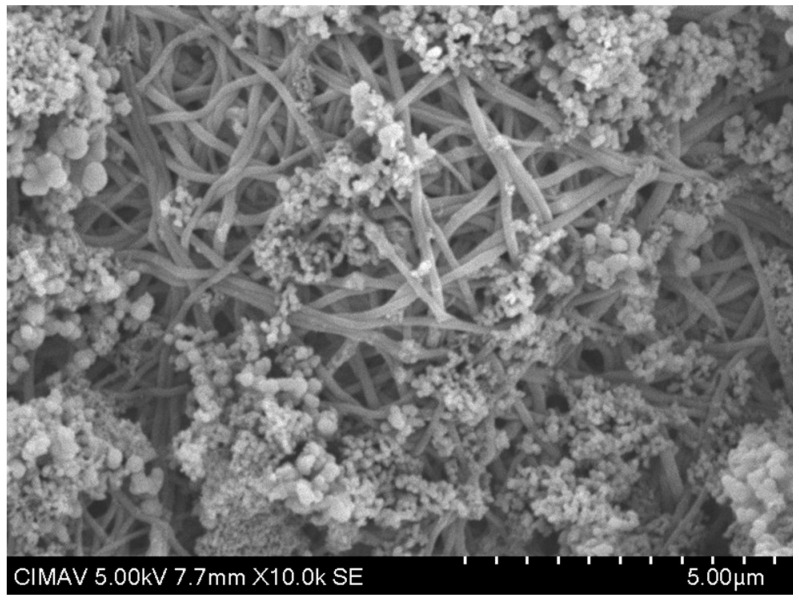
SEM image of TiO_2_-PVA-HAp in solution.

**Figure 14:**
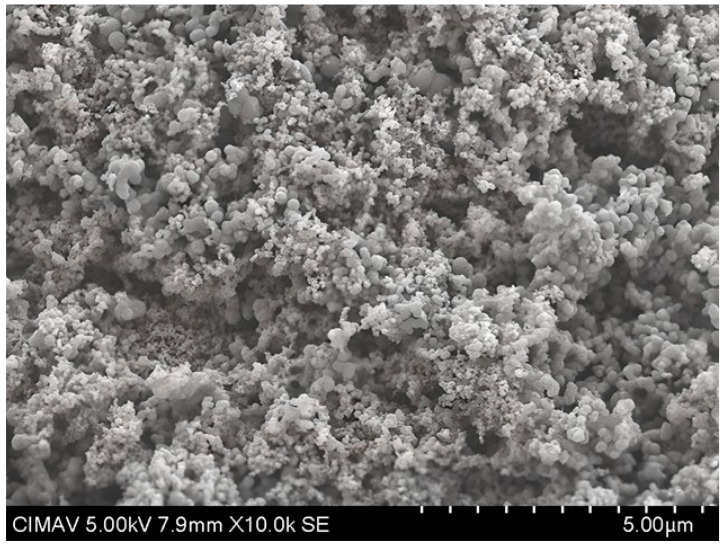
SEM micrograph of TiO_2_-PVA-HAp at 700 °C.

**Figure 15:**
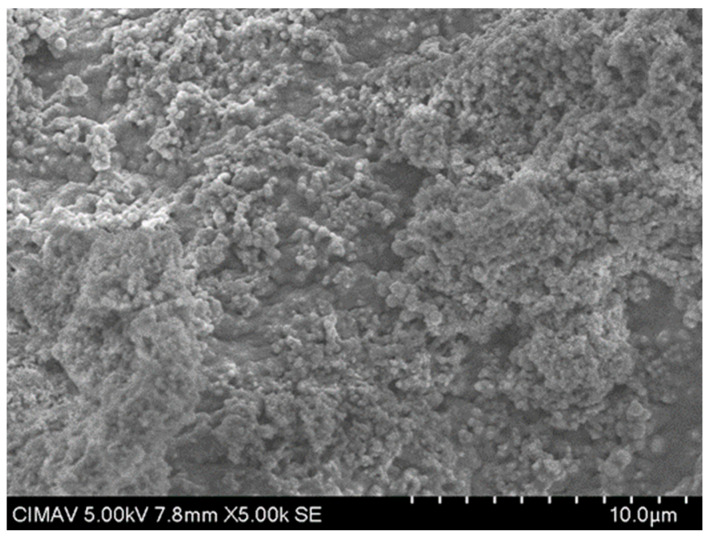
SEM micrograph of TiO_2_-PVA-HAp at 400 °C.

**Figure 16:**
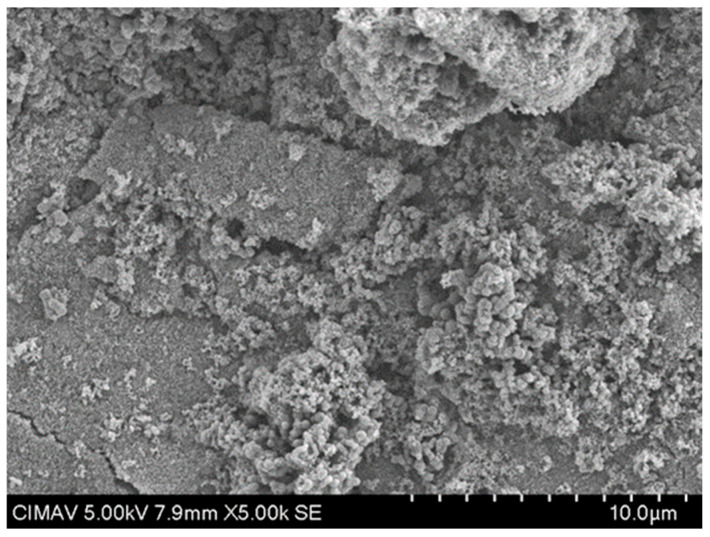
SEM micrograph of TiO_2_-PVA-HAp at 600 °C.

The results in Figure 13 show the HAp-TiO_2_ nanofibers in solution before being calcined at 700 °C; the hydroxyapatite nanofibers and the beginning of the formation of particle clusters with a diameter range of 100–500 nm are clearly observed. After the formation of the gel containing the Hap-TiO_2_ nanofibers, it was calcined at a temperature of 700 °C. In Figure 14, one can see the complete formation of particles. In this image, the hydroxyapatite nanofibers are not seen. This behavior in the change in morphology reveals the mechanism of molecular self-assembly through the Columbian interaction. The participation of the Ca^2+^ particles found in the hydroxyapatite, positively charged, and the Ti-O bonds of the TiO_2_, negatively charged, allows for the nucleation of the hydroxyapatite, forming supramolecular conglomerates of spherical shape. In this way, by increasing the temperature, it is possible to favor the growth velocity of the particles, taking advantage of the physicochemical characteristics of hydroxyapatite, such as pyrolytic activity and thermal stability. Furthermore, treatments were conducted at 400 and 600 °C. The formation of spherical structures is depicted as conglomerates in Figure 15 and Figure 16. Some strategies for the formation of structures related to the use of TiO_2_-HAp have been proposed in various areas such as the development of biointerfaces, tissue engineering, biomedical applications, and others [43,44,45].

**Figure 17:**
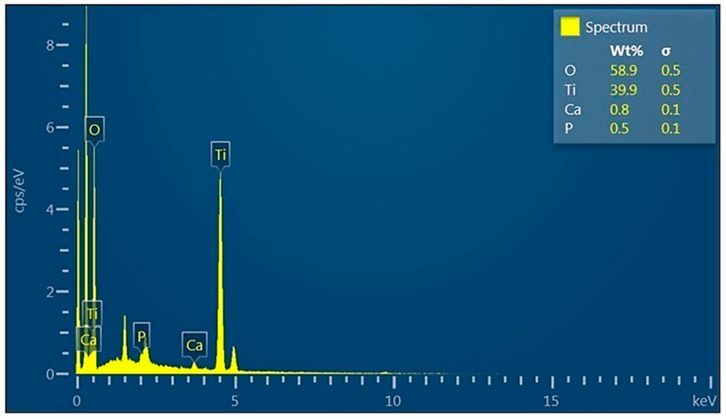
Energy dispersive X-ray analysis of nanocompounds.

Figure 17 shows the energy dispersive X-ray analysis, which was used to identify the composition of the chemical elements present in the sample, finding O, Ti, Ca, and P, as well as their respective weight percentages. This demonstrates the interaction of the hydroxyapatite and titanium nanofibers.

## 4. Conclusions

In this research, we have reported the formation of nanocompounds of TiO_2_ using PVA-HAp nanofibers with a range of 100–500 nm in diameter, respectively. The crystalline phase of the nanocompounds was confirmed by X-ray analysis. The nanocompounds were analyzed to establish the supramolecular interaction of HAp nanofibers in the presence of TiO_2_. In this manner, we propose that the electrostatic interaction and biocompatibility are very important in the formation of these structures because we study the functionality of several characteristics, such as isotropy, electronic frequency, thermal activity, and specificity. The coefficient of determination R2, with a value of 1, allows us to establish a prediction mechanism in the formation of this type of structure using nanofibers of HAp and TiO_2_. The chemical activity and the stability of the nanocompounds were confirmed by X-ray analysis. The significance of the temperature effect on electrostatic interaction during the design of supramolecular structures is indicated by the cluster formation behavior observed in SEM analysis at 400 and 600 °C. On the other hand, with this method, it would be possible to study several events such as electronic interaction, molecular self-assembly, physicochemical characteristics, catalysis, and thermodynamic effects. Beyond these aspects, the potential uses of the nanocompounds may extend not only to biomedical and environmental applications but also to construction and urban contexts, for instance, in coatings and materials that contribute to sustainable built environments.

## Data Availability

Data are contained within this article. For more information, one can contact the corresponding author.
